# A novel segmental absence of intestinal musculature with small intestinal stenosis: a case report

**DOI:** 10.1186/s12876-020-01419-4

**Published:** 2020-08-17

**Authors:** Kosuke Kashiwagi, Keisuke Jimbo, Kenji Hosoi, Go Miyano, Takahiro Kudo, Atsuyuki Yamataka, Toshiaki Shimizu

**Affiliations:** 1 Department of Pediatrics, Juntendo University Faculty of Medicine, 2-1-1 Hongo, Bunkyo-ku, Tokyo, 113-8421 Japan; 2 Department of Pediatric General and Urobenital Surgery, Juntendo University Faculty of Medicine, 2-1-1 Hongo, Bunkyo-ku, Tokyo, 113-8421 Japan

**Keywords:** Crohn’s disease, Creeping fat sign, Power Doppler, Ultrasonography

## Abstract

**Background:**

Segmental absence of intestinal musculature (SAIM) is a rare cause of intestinal obstruction and perforation due to partial or complete defects in the intestinal muscularis propria in neonates and is occasionally observed in adulthood.

**Case presentation:**

The first case of small intestinal stenosis derived from SAIM, which was difficult to differentiate from Crohn’s disease (CD), is reported. A 4-year-old girl presented with abdominal pain, anemia, and a positive fecal occult blood test. She was initially diagnosed with CD and started on treatment. Because her gastrointestinal symptoms persisted, her previous pediatricians tried to carry out capsule endoscopy, but it was not possible because the patency capsule was retained. Therefore, she was referred to our institute and re-evaluated. The patency capsule examination was repeated to re-evaluate small intestinal passage, but it stagnated again. Abdominal ultrasonography showed a poorly deformable intestinal tract that narrowed rapidly from the dilated segment and had a thin wall with an irregular laminar structure. In addition, unlike the typical ultrasonic CD findings, the power Doppler signal enhancement at the intestinal wall and “creeping fat sign” were not found. The patient was referred for laparoscopic observation to pediatric surgeons, who confirmed a prominently dilated intestinal tract 40 cm proximal to the ileocecal valve, which was resected. Histopathological findings showed longitudinal muscle hypoplasia of the resected, dilated intestinal tract and fat replacement of the muscle layer. At the stenosis site, the muscle layer was fibrotic and showed incomplete muscle arrangement. Because of these findings, she was diagnosed with SAIM. After the surgical treatment, no gastrointestinal symptoms relapsed, and the fecal occult blood test has remained negative for 2 years. Moreover, 8 months after surgery, double-balloon endoscopy showed no abnormalities, such as a longitudinal ulcer and cobblestone appearance.

**Conclusions:**

In the present case, SAIM involved not only intestinal ileus and perforation, but also small intestinal stenosis. Although no other reports have demonstrated the usefulness of abdominal ultrasonography for the diagnosis of SAIM, the present report suggests that ultrasonography may be useful for differentiating SAIM from CD by close observation of the area around the small intestinal stenosis.

## Background

Segmental absence of intestinal musculature (SAIM) is a rare disease associated with intestinal obstruction and perforation in all ages due to partial or complete defects in the intestinal muscularis propria [1]. In 1967, SAIM was initially reported as a cause of intestinal obstruction in neonates [2, 3]. It was known as a phenomenon of spontaneous focal perforations of the intestinal tract or ileus without clinical and histological features of necrotizing enterocolitis [4]. SAIM was often diagnosed after intestinal obstruction and perforation in newborns, but some cases were diagnosed in adulthood with minor symptoms in childhood [3, 5–7]. The intestine without its muscularis propria developed intestinal wall thinning and hypoperistalsis [8], and most of the muscle defect portion of the intestine had no Auerbach and Meissner plexuses [9]. To the best of our knowledge, cases of small intestinal stenosis derived from SAIM have not been reported. A case of a child with small intestinal stenosis due to SAIM, in which ultrasonography played an important role in differentiating SAIM from Crohn’s disease (CD), is reported.

## Case presentation

A 4-year-old girl with a past history of Kawasaki’s disease at 1 year of age developed abdominal pain and anemia (hemoglobin: 4.8 g/dL) with a positive fecal occult blood test. Her family had no apparent medical history. She was therefore referred to a local hospital by her doctor. Abdominal contrast computed tomography on admission showed focal small intestinal wall thickening and dilatation, and several un-cascaded small ulcers were detected by colonoscopy at the terminal ileum, and she was diagnosed as having CD. She was started on 5-aminosalicylic acid (5-ASA) and partial enteral nutrition which means the patient receives 50% of her calories from the elemental diet, and the remainder from the low-fat diet, but her abdominal pain persisted. For further evaluation of the small intestine, upper gastrointestinal and small bowel series was performed using X-ray contrast media was performed, but no obvious abnormalities could be confirmed. Therefore, previous pediatricians attempted carry out capsule endoscopy, but its feasibility was uncertain due to retention of the patency capsule (PC). In addition, adult gastroenterologists were consulted in an attempt to perform small intestinal double-balloon endoscopy, but due to technical difficulties the intestinal lesion could not be targeted.

**Fig. 1:**
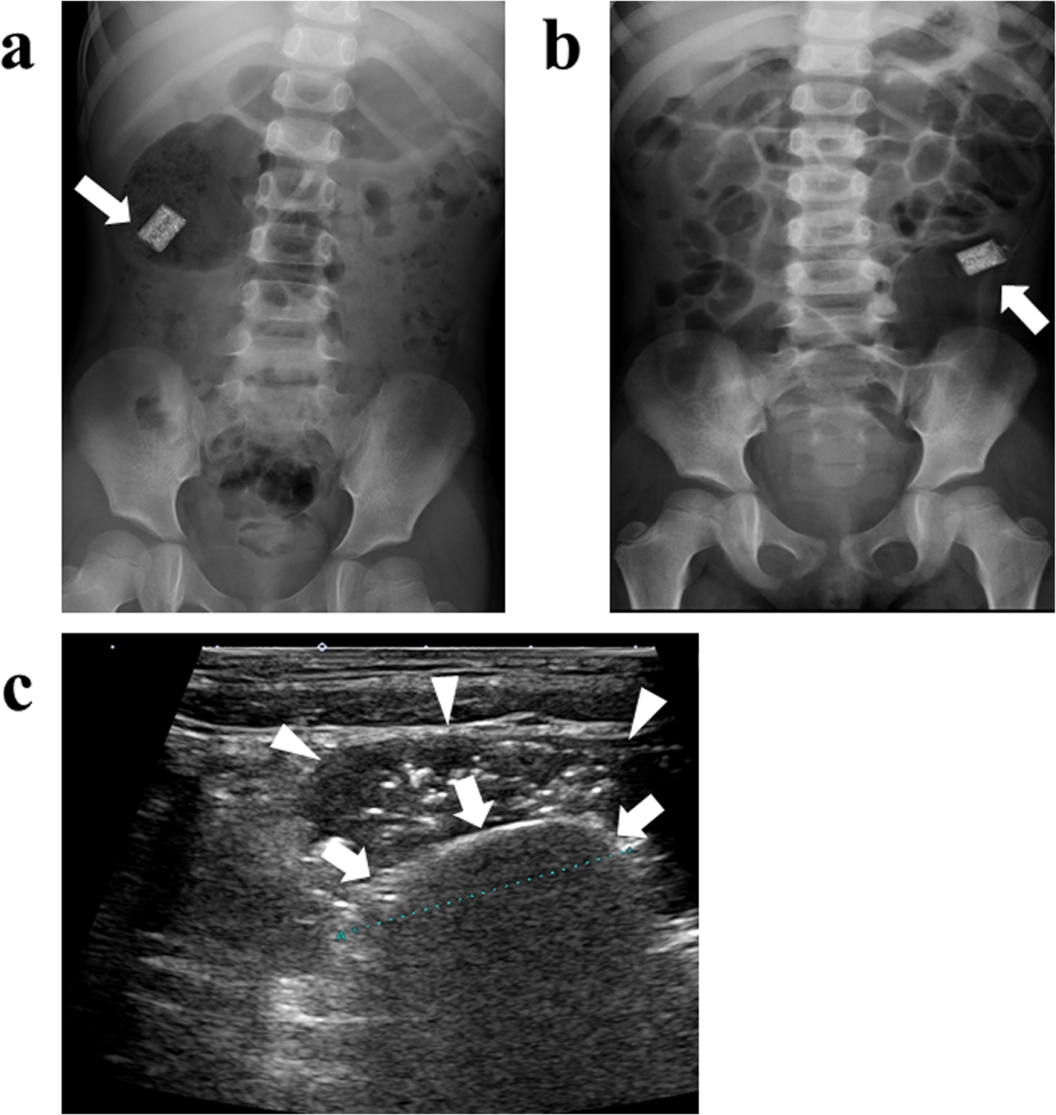
Patency capsule stagnation (arrow) at the dilated small intestinal tract. **a** shows the abdominal X-ray in the standing position; **b** shows the abdominal X-ray in the supine position; **c** shows stagnation of the patency capsule (arrow) at the dilated small intestinal tract (arrowhead)

**Fig. 2:**
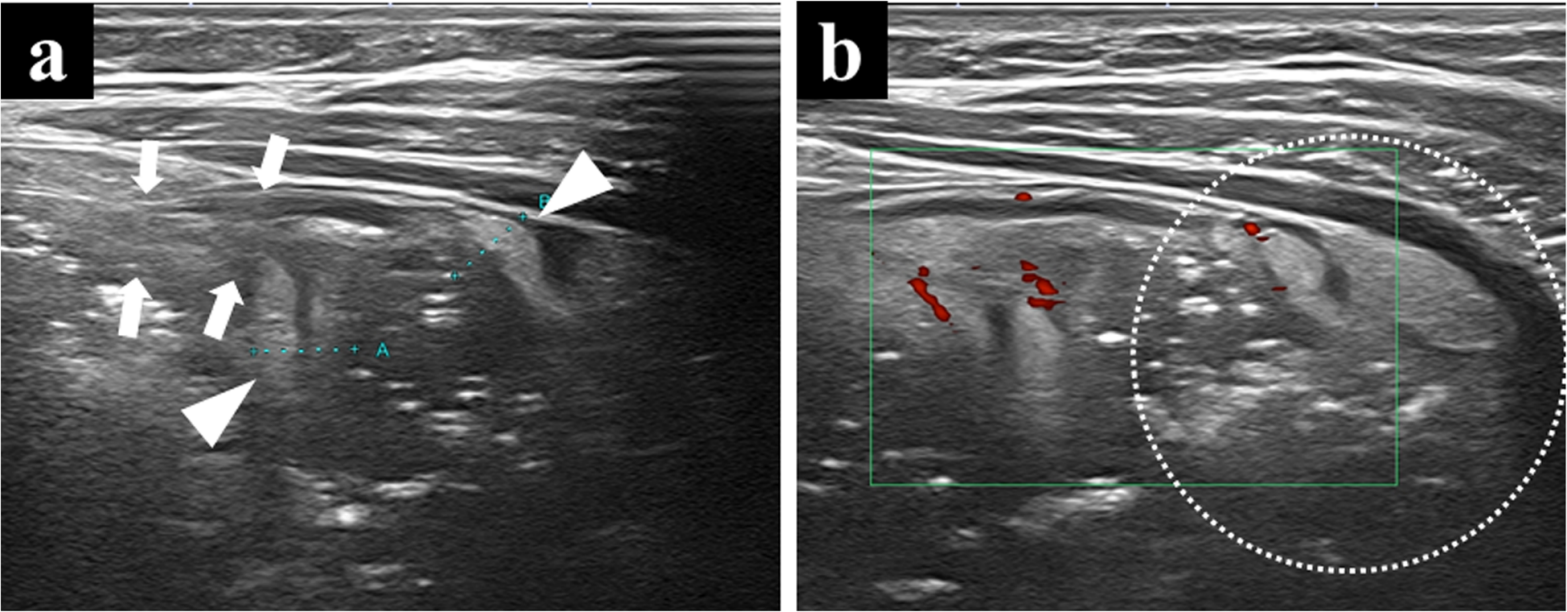
Abdominal ultrasonic findings of the intestinal lesion with a high-frequency 11-MHz linear transducer. **a** shows the poorly deformable intestinal tract, which narrows rapidly (arrow) from the dilated segment (arrowhead) and has a thin wall with irregular laminar structure; **b** shows the peripheral side of the focally narrowed intestinal tract that has no power Doppler signal enhancement and no “creeping fat sign” around the lesion

**Fig. 3:**
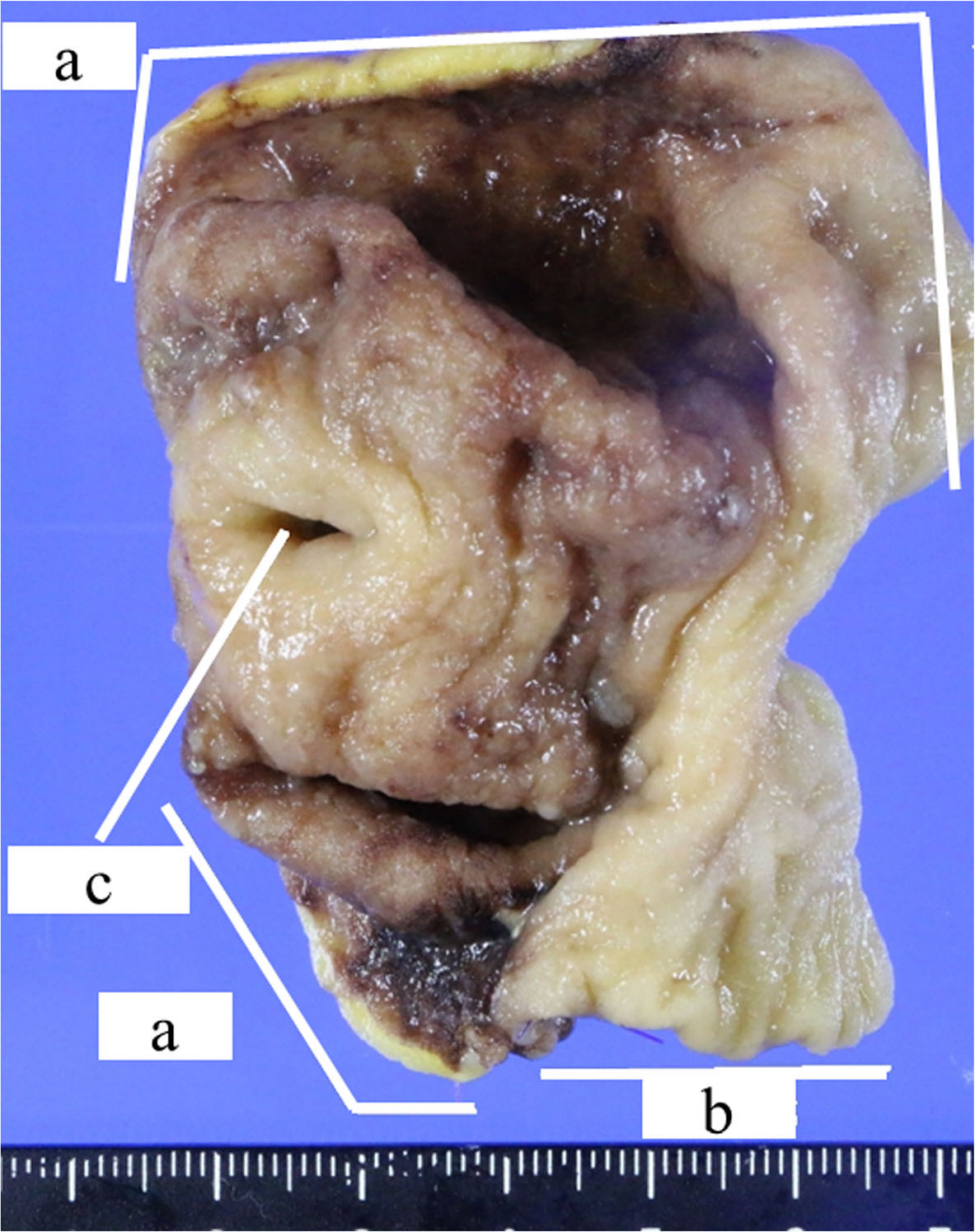
Macroscopic findings of the resected intestinal tract. **a** shows the dilated portion with the thin intestinal wall; **b** shows normal small intestine; **c** shows the peripheral side of the intestinal stenosis of 7 mm in diameter with thickening of the surrounding wall through which the patency capsule (26 mm × 11 mm) cannot pass

**Fig. 4:**
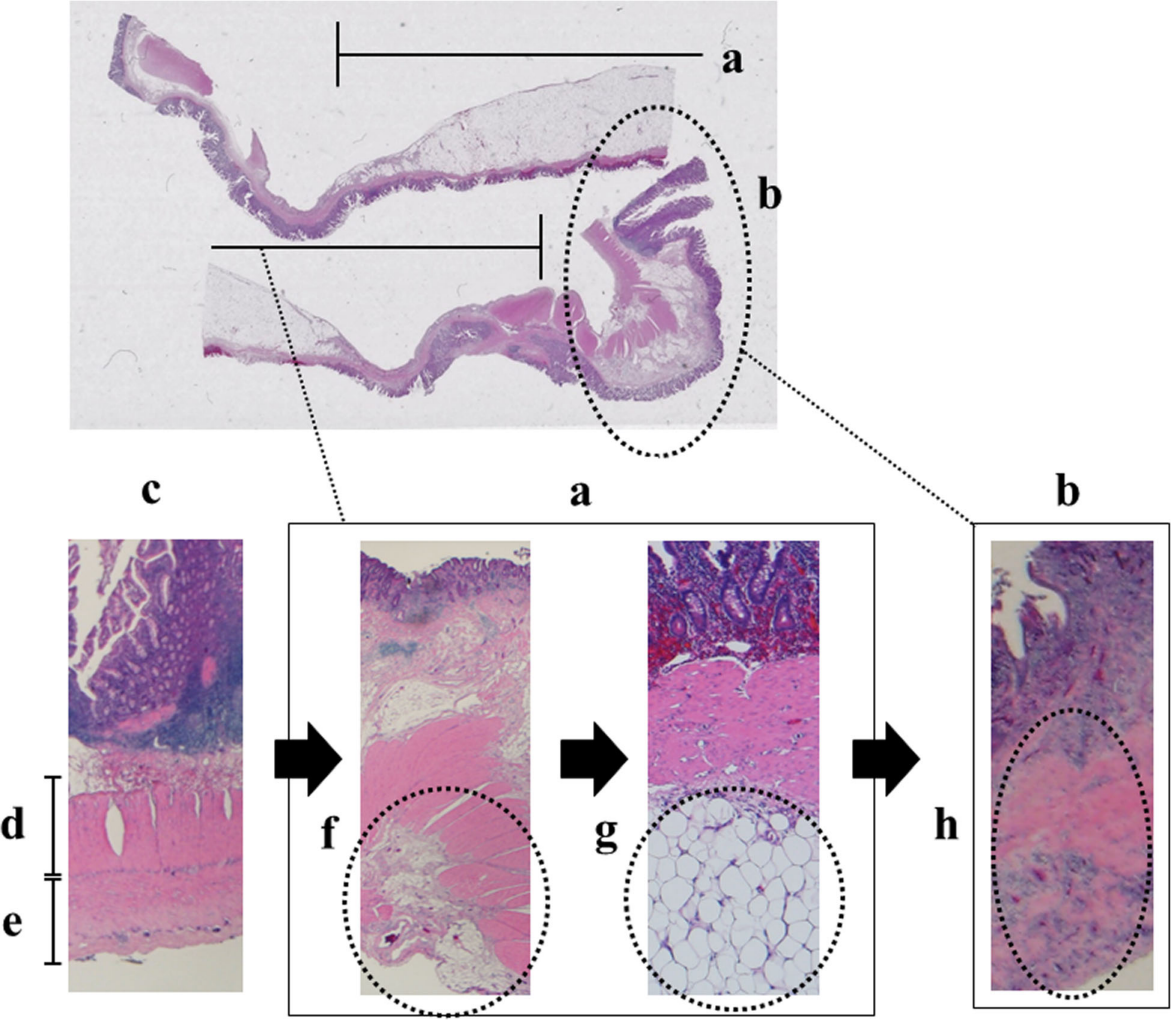
Histopathological findings of the resected intestinal tract. **a** shows the segment of intestinal dilation; **b** shows the segment of intestinal stenosis; **c** shows a normal intestinal portion; **d** shows the normal circular muscle layer; **e** shows the normal longitudinal muscle; **f** shows hypoplastic longitudinal muscle in the dilated portion; **g** shows adipose tissue replacing hypoplastic muscle in the dilated portion; **h** shows the incomplete muscle layer with fibrosis in the intestinal stenosis

Therefore, in 6 years old, the patient was referred to our institute for re-evaluation of her illness. Anthropometric measurements included height and weight were 111.4 cm (− 0.4 standard deviation) and 18.1 kg (− 0.6 standard deviation), respectively, suggesting no growth failure was apparent. On physical examination, no remarkable abnormalities were found. Laboratory examinations at that outpatient visit showed the hemoglobin level improving from 4.8 g/dL to 12.1 g/dL, C-reactive protein level of 0.05 mg/dL, and fecal occult blood of 189 ng/mL (normal range, 0–80 ng/mL). A positive fecal occult blood finding indicated re-evaluation with ileoscopy and total colonoscopy with biopsy, but there were no abnormal findings. Patency capsule was re-performed, to re-evaluate the obstruction of small bowel transit, but it retained again. At that time, abdominal X-ray and ultrasonography showed dilatation of the small intestine with the PC’s stagnation. (Fig. 1). Abdominal ultrasonography demonstrated a poorly deformable intestinal tract, which narrowed rapidly from the dilated segment and had a thin wall with an irregular laminar structure. In addition, unlike the typical ultrasonic CD findings, power Doppler signal enhancement at the intestinal wall and the “creeping fat sign” [10] in the surrounding area of the intestinal wall were not found (Fig. 2). Although *SLCO2A1* sequencing was performed to differentiate this from chronic enteropathy associated with *SLCO2A1*, in which small intestinal stenosis forms with the healing of multiple ulcers [11], no mutations could be detected. The patient was referred to pediatric surgeons for laparoscopic observation, and they confirmed a prominently dilated intestinal tract (47 × 47 × 30 mm) 40 cm proximal to the ileocecal valve, which was resected. Macroscopically, the resected intestinal tract had a thin wall and stenosis of 7 mm in diameter, with thickening of the surrounding intestinal wall at the proximal portion. (Fig. 3). Histopathological findings showed longitudinal muscle hypoplasia of the resected dilated intestinal tract and fat replacement of the muscle layer. At the stenosis site, the muscle layer was fibrotic and showed incomplete muscle arrangement (Fig. 4). Because of these findings, she was diagnosed with SAIM. After the surgical treatment, the patient presented with no adverse event from surgery and no gastrointestinal symptoms relapsed. Fecal occult blood test remained negative without the use of 5-ASA and partial enteral nutrition for 2 years. Moreover, 8 months after surgery, double-balloon endoscopy showed no abnormalities, such as a longitudinal ulcer and cobblestone appearance.

## Discussion and conclusions

Although the cause of the small intestinal intrinsic muscle layer loss in SAIM remains unknown, congenital or acquired factors have been considered. Most cases of congenital SAIM were reported in preterm and low-birthweight infants, and some researchers hypothesised that SAIM was caused by dysplasia of the small intestinal muscle layer in the embryonic period or as a result of transient ischemia after birth [5, 6, 8, 9]. In particular, typical congenital SAIM presented clinically as intestinal perforation or obstruction in neonates and early childhood. In contrast, acquired SAIM cases in adulthood were reported to have intestinal ischemia secondary to hypertension or chronic constipation involved in the onset of acquired SAIM [12, 13]. Our present patient presented with no past history of hypertension and chronic constipation, hence a diagnosis of congenital SAIM was confirmed.

According to adult and pediatric case reports and systematic reviews from 1997 to 2016 identified in a PubMed search for segmental absence of intestinal musculature, 2, 16, and 8 cases occurred in the duodenum, small intestine (7 cases: jejunum, 9 cases: ileum), and colon (6 cases: sigmoid colon, 1 case: ascending colon, 1 case: descending colon), respectively. These SAIM cases were generally diagnosed after emergency surgery due to intestinal perforation [6, 7, 12–18]. Although the present case involved the ileum, which was a relatively typical site in previous reports, cases with small intestinal stenosis that were diagnosed before intestinal perforation have not been previously reported. In addition, there have been no published papers reporting ultrasonic imaging findings of SAIM, including cases in which ultrasonography contributed to the diagnosis, as in the present case.

Although SAIM is characterized by thinning of the intestinal tract due to histological hypoplasia or loss of the inner muscular layer, but maintenance of the mucosal layer, a case in which the muscle defect portion of the intestinal wall was replaced by fibrous tissue has been reported [7]. The histopathology of the present case also showed the same findings as previously reported, and thus some recurrent inflammatory damage in the dilated intestinal tract may have resulted in scarring and fibrosis during repeated mucosal healing of the ulcerous lesion and small bowel stenosis. Furthermore, in the present case, there appeared to be intestinal congestion in dilated intestine, which caused ulcerative bleeding and anemia.

On ultrasonic examination, it was reported that ultrasonic Doppler signal enhancement of the thickened intestinal wall [19] and hyperechoic thickening of extraintestinal stromal tissue, called the “creeping fat sign” [10], were considered to be essential findings for making a diagnosis of CD. In contrast, no power Doppler signal of the thickened intestinal wall with high echogenicity might suggest intestinal stenosis. The present case had intestinal stenosis similar to the ultrasonic findings of CD, but neither Doppler signal enhancement nor the “creeping fat sign” around it. Therefore, to differentiate SAIM and CD by ultrasonography, it might be useful to identify atypical CD findings around the stenotic lesion, but further work is needed in larger cohorts. In addition, in some SAIM cases, it was reported that they had other lesions of SAIM within the intestinal tract [1], but double-balloon endoscopy confirmed no other SAIM lesions in the present case.

In conclusion, a novel SAIM case with small intestinal stenosis that was difficult to differentiate from typical CD was reported. The SAIM involved not only intestinal ileus and perforation, but also small intestinal stenosis. Although no other reports have demonstrated the usefulness of abdominal ultrasonography for the diagnosis of SAIM, this report suggests that ultrasonography may be useful for differentiating SAIM from CD by close observation of the area around the small intestinal stenosis.

## Data Availability

All data generated or analyzed during this study are included in this published article.

## Abbreviations

CD: Crohn’s disease
5-ASA: 5-aminosalicylic acid
PC: Patency capsule
SAIM: Segmental absence of intestinal musculature
